# Analysis of liquid–vapor mixed migration mechanism in unsaturated soil based on the effect of temperature on soil microstructure

**DOI:** 10.1038/s41598-023-47985-x

**Published:** 2023-11-23

**Authors:** Jianxun Zhang, Xuesong Mao

**Affiliations:** Highway Academy, Chang’an University, Xian, 7100000 People’s Republic of China

**Keywords:** Civil engineering, Environmental impact, Hydrogeology

## Abstract

Moisture migration in unsaturated soils is a result of the interaction between temperature and soil microstructure. In order to reveal the mechanism of moisture increase of subgrade soils under diurnal cycle conditions, a series of macro and microscopic tests were carried out on the unsaturated silty clay and sand soil, including liquid–vapor mixed migration tests simulating a one-dimensional subgrade, environmental scanning electron microscope (ESEM), and matrix suction test. Then, the soil microstructure in microscopic images was investigated using the particle (pores) and cracks analysis system (PACS). Next, the relationship between the thermal effects of the soil–water characteristic curve (SWCC) and changes in soil microstructure was analyzed. Finally, the change mechanism of liquid–vapor mixed migration based on the change in soil microstructural under thermal effects was analyzed. The results showed under the diurnal cycle, both the silty clay and sand soil columns appeared in the phenomenon of a “diurnal cycle of water vapor migration”, which led to moisture accumulation at the top of the soil layer. In silty clay soil column, moisture was primarily driven by water vapor pressure and migrated upwards. Additionally, moisture redistribution led to changes in soil microstructure, which in turn influenced the process of moisture migration. The moisture content in the upper soil layer increased making both inter-aggregate and intra-aggregate pores decrease. The moisture content in the lower soil later decreased, leading to the water-holding capacity of the lower soil layer to increase. So, the moisture migration gradually decreased at night. In the sand soil column, moisture migration was mainly driven by gravity potential and migrated downwards. Moisture redistribution made inter-aggregate pore and matrix suction of the upper soil layer increase, leading to an increase in moisture migration at night.

## Introduction

In the arid and semi-arid areas of Northwest China, excessive moisture accumulation has been discovered between the inorganic binder layer and subgrade of various transportation facilities such as highways, railways, and airport roads^1–3^. This phenomenon resulted in cracks and deformation on the pavement, frost heaving, and uneven settlement on the subgrade, severely affecting the normal operation of the road^4,5^. Current research indicated that this moisture increase was primarily caused by the moisture migration in soil under the action of temperature gradient and got blocked by the impermeable inorganic binder layer, ultimately accumulating under the covering layer^3,6^. Virtually, the moisture migration of soil was essentially caused by the interaction between temperature and moisture in soil structure. In this region, subgrade soil is typically found as unsaturated soil, which the soil structure mainly refers to the arrangement and distribution of soil particles (pores) and the interaction between moisture, particles (pores), and the gas phase. On the one hand, the pore structure of soil is the channel of moisture migration (liquid water and water vapor). On the other hand, the soil structure is influenced by temperature determining the moisture driving force. Therefore, exploring the relationship between temperature and soil structure is crucial to reveal the mechanism of moisture migration, which also holds significant practical implications for the prevention and control of road engineering disasters.

Numerous scholars have studied the moisture migration of unsaturated soils, focusing on moisture migration properties, moisture driving force, and its constitutive relationship. Ultimately, the study of moisture migration was the study of the relationship between soil structure and temperature. Among them, the effects of initial moisture content, temperature gradient, dry density, soil type, and test time on moisture migration have been widely explored. For example, Wang et al. examined how initial moisture content affected moisture migration^7^. They proposed that connected pores in unsaturated soils were necessary for liquid and water vapor migration. When the moisture content was too high, a large amount of liquid water led to the disconnection of soil pores, blocking the channels for moisture migration and hindering the movement of water vapor migration. Zhang et al. visually analyzed the mechanism of water vapor migration in coarse-grained soil using tracer tracking and image processing technology^2^. They emphasized the importance of considering the migration of moisture vapor in unsaturated subgrades, as it was a major factor leading to frost heave in coarse-grained soil fillers in cold and arid areas. Apart from that, Li et al. summarized the mechanism that caused disasters due to moisture accumulation under the covering layer and proposed the concept of the “pot-cover effect”^3^. Teng et al. classified “pot-cover effect” into two types based on whether the shallow soil was frozen^8^. Zhang Ruru^9^, Banimahd^10^, and Song Erxiang^11^ established mathematical models and used the finite element method to simulate the “canopy effect”. In addition, the change in temperature has a significant influence on the moisture driving force of soil structure. The relationship between soil particles and water in pores is represented by the soil–water characteristic model, which is the fundamental constitutive model for studying the migration of liquid water. Numerous scholars have confirmed that the soil–water characteristic model had a thermal effect^12,13^. When influenced by a temperature gradient, liquid water will migrate from the high-temperature section to the low-temperature section. Furthermore, William Thomson discussed the relationship between water vapor pressure, temperature, and matrix suction in unsaturated soil and proposed the Kelvin formula for calculating moisture vapor pressure (water vapor driving force) in soil pores. It can be seen from the previous studies that the moisture migration of unsaturated soil was mainly studied from the perspective of macro soil structure change. In fact, the moisture migration of unsaturated soil was the macroscopic manifestation of the change in soil microstructure. Exploring the microscopic soil structure is helpful in revealing the relationship between water vapor migration and soil structure. It also helps to understand the change mechanism for the thermal effect of the soil-moisture characteristic model. This can provide a better explanation for the moisture migration mechanism of unsaturated soil. Therefore, studying the evolution mechanism of microstructure in unsaturated soil with temperature plays a crucial role in revealing the moisture migration process.

Microstructural studies were based on the rational and accurate acquisition of qualitative information on soil microstructure, and so far, various means including the mercury intrusion method, computed tomography (CT), and imaging techniques have been introduced to achieve this goal. Among all the technologies used for studying soil microstructure, scanning electron microscope (SEM) was the most widely used. However, there were certain limitations to its application. For example, non-conductive samples required conductivity enhancement treatment, strict vacuum conditions were necessary, and the presence of moisture in the sample was not allowed. In the 1960s, the environmental scanning electron microscope (ESEM) was developed based on the traditional SEM. It allowed direct observation of moisture-containing or insulating samples, which helped to maintain the natural state of the samples and reflect the real surface structure details of the materials^14^. In the field of civil engineering, ESEM have been widely used to observe the microstructure of the hydration process of cement^15^, and the hydration/dehydration cycle of expansive soil^16,17^. Moreover, the core issue in the study of soil microstructure lies in dealing with the quantitative description of microstructure characteristics, such as the number and diameter of pores. PCAS software can integrate image processing, statistics, and analysis, which can effectively identify and extract the required soil microstructure information from electron microscopy images^18^. Therefore, the combination of PCAS software and ESEM technology can achieve optimal results in the analysis of the microstructure of unsaturated soil.

Currently, most studies focus on the moisture migration of unsaturated soil in the negative temperature season. It has been confirmed that the migration of liquid–vapor mixed in the soil freezes into ice, resulting in a significant increase in moisture content at the surface and freezing front^2,19,20^. In arid and semi-arid areas, where the temperature difference between day and night is substantial, the soil experiences intense water vapor migration and frequent increases in moisture content in the shallow soil^21,22^. Hence, it is also crucial to study the moisture migration of subgrade covered with the impermeable inorganic binder layer under diurnal cycles during the positive temperature season. The purpose of this study is to reveal the evolution mechanism of moisture migration in unsaturated soil under the diurnal cycle from the perspective of soil microstructure change. Therefore, the moisture migration test of the unsaturated soil simulating subgrade working conditions firstly was conducted. Next, the intrinsic microstructure of the unsaturated soil under different temperature conditions was observed using ESEM, and the soil microstructure was quantitatively analyzed using PCAS image processing software. After that, the matrix suction test considering the thermal effect was conducted. Then the relationship between the thermal effects of SWCC and changes in soil microstructure was analyzed. Finally, the mechanism of moisture migration in the subgrade soil under the diurnal cycle was revealed from the perspective of the interaction between temperature and the soil microstructure. This study is of great significance to improve the moisture migration theory of unsaturated soil.

## Experimental scheme and tests

### Materials

**Figure 1:**
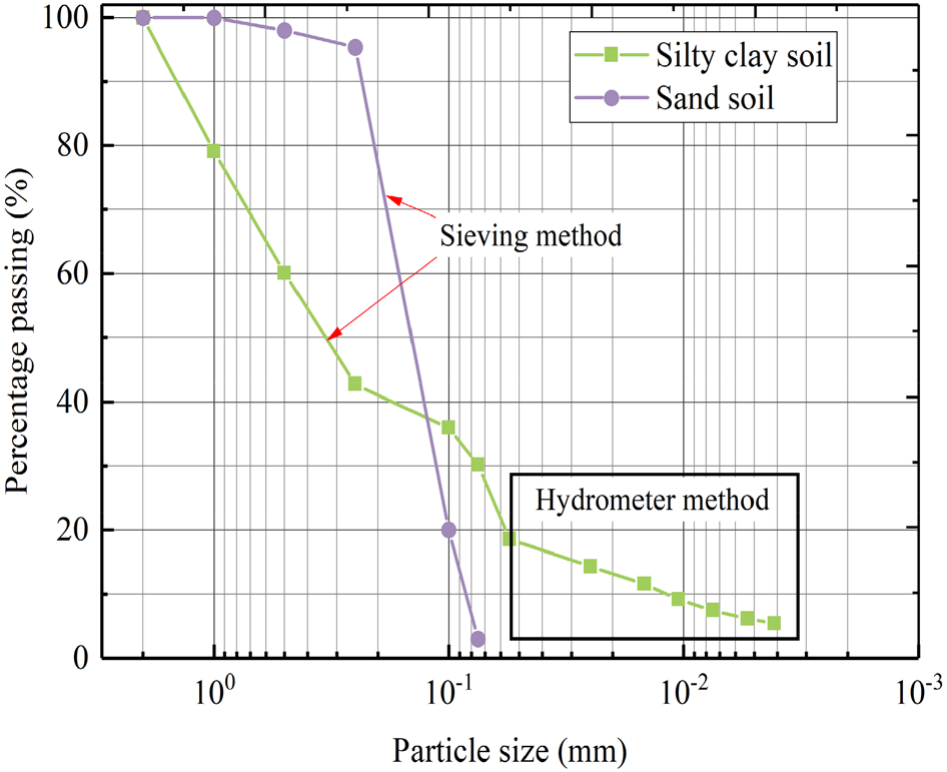
Particle size distribution.

The soil materials (silty clay and sand) used in this study were collected in Yanan County, Shaanxi Province. Table 1 lists the basic physical parameters of the studied soil measured according to the Chinese standard for geotechnology testing method (GB/T 50123-2019). The specific gravity, compaction characteristics, and limit moisture content were measured according to the pycnometer method, the standard compaction tests, and the liquid-plastic limit combined method. In addition, the distribution with a particle size greater than 0.075 mm was analyzed by the sieve method, while the distribution with a particle size less than 0.075 mm was analyzed by the hydrometer method, as shown in Fig. 1.

**Table 1:** Physical properties of the studied soils.

Soil type	Specific gravity	Limit moisture content (%)		Compaction characteristic	
		Liquid limit	Plastic limit	Optimum moisture content (%)	Maximum dry density (g/cm^3^)
Silty clay	2.72	30.50	17.69	14.1	1.86
Sand	2.67	/	/	9.0	1.90

### Moisture migration test

**Figure 2:**
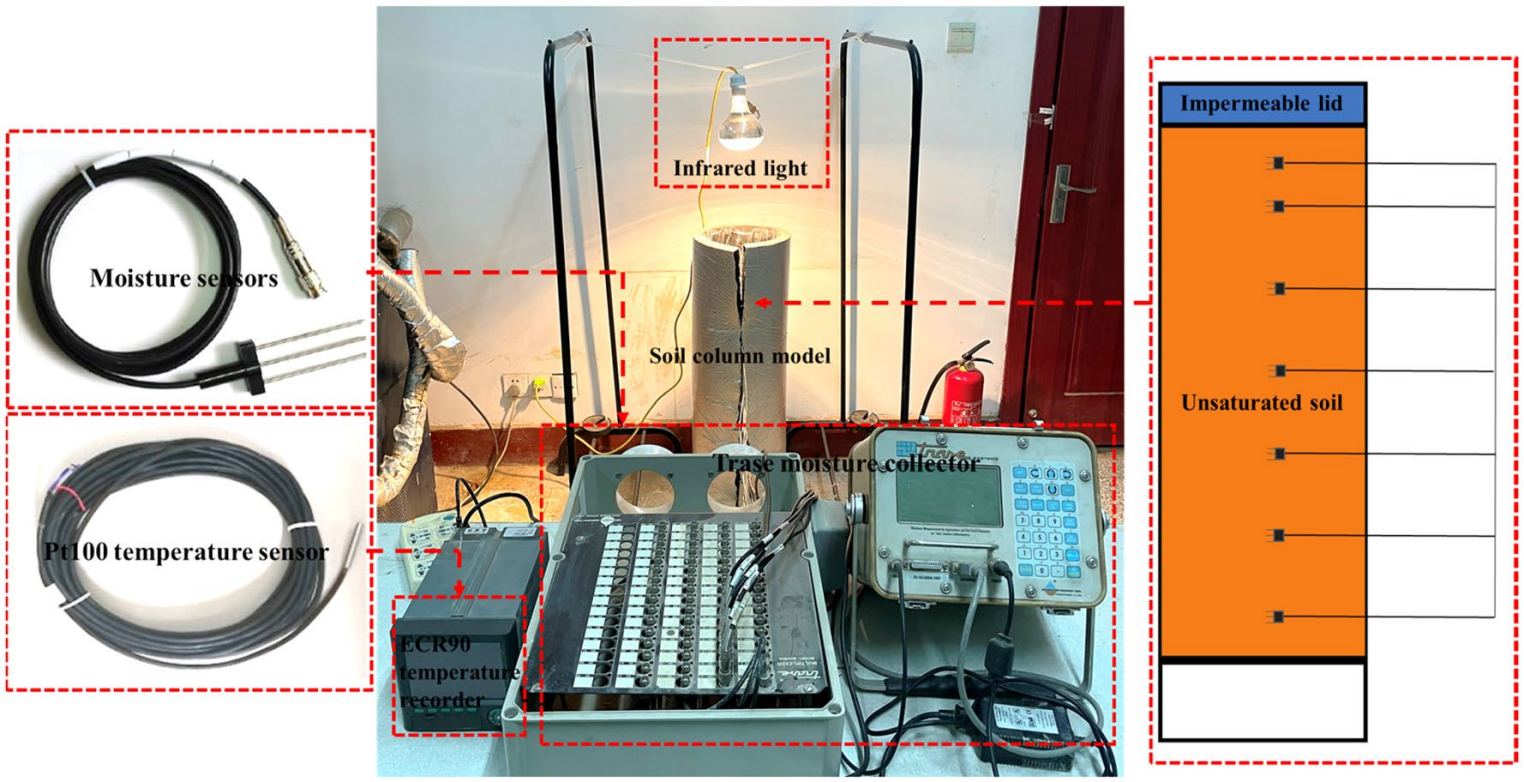
Moisture migration test devices.

To reveal the liquid–vapor mixing migration mechanism of the subgrade soil under multiple diurnal cycles, moisture migration tests of one-dimensional soil columns were performed for silty clay soil and sand soil. A one-dimensional soil column model apparatus simulating the subgrade working conditions was designed, as seen in Fig. 2. The device mainly consisted of soil column models, a data logging system, and an infrared light. The soil column models consisted of a specimen tube (height: 65 cm, inner diameter: 20 cm) made of transparent Plexiglas material and an impermeable lid to simulate the one-dimensional subgrade covered with the inorganic binder base. The data logging system that measured temperature and moisture data consisted of an ECR90 temperature recorder, a MiniTrase moisture collector, and several temperature and moisture sensors. The temperature and humidity data of the soil column were recorded and saved in real-time by the sensor into the recorder. ECR90 temperature recorder can simultaneously collect 16 channels of data with an accuracy of 1 °C. And PT100 soil temperature sensor was made of a high-precision thermistor, its working temperature range was − 70 °C–150 °C. The measuring range of the Trase moisture collector was 0–100% with an accuracy of 2%. The probe of the TDR moisture sensor was stainless steel material, and its working temperature range was − 30 °C–60 °C. First, the soil was filled in the soil column in layers and controlled the compaction degree of 100%. The temperature and moisture sensors simultaneously were placed at soil column depths of 5 cm, 10 cm, 20 cm, 30 cm, 40 cm, 50 cm, and 60 cm when filling the soil columns. After the soil filling was completed, the impermeable lid was covered on the soil column. Subsequently, the infrared light simulating sunshine conditions was arranged on the top of the soil column models. The highest temperature at the top of the soil column was controlled at 45 °C by adjusting the distance between the light and the soil column. A day-night temperature cycle (24 h) was controlled, with 14 h of light on to simulate daytime sunshine conditions and 10 h of light off to simulate nighttime conditions. Finally, the test device was housed in a thermostatic chamber to ensure that external temperature variations did not affect the test.

### ESEM observation

**Figure 3:**
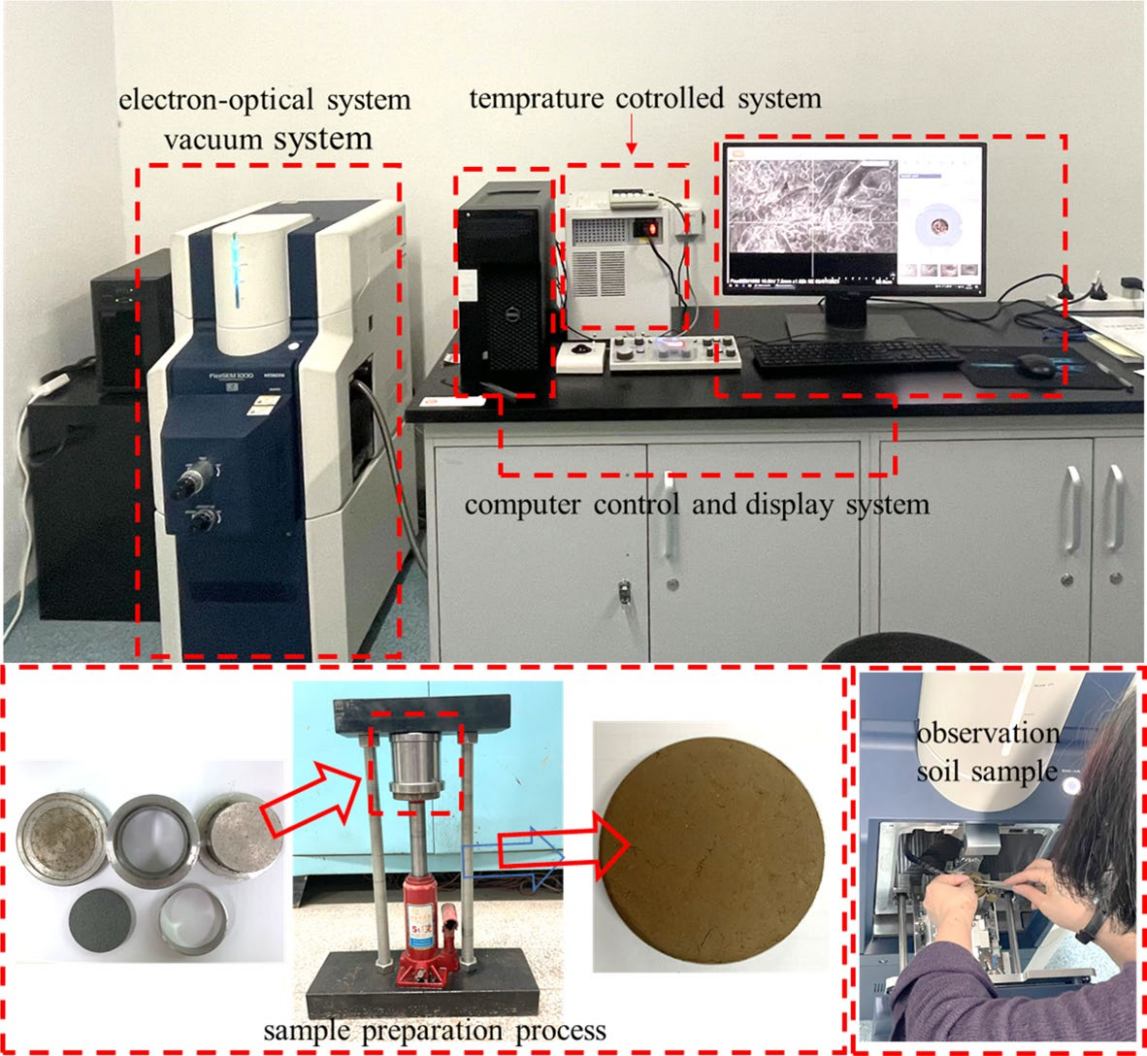
ESEM observation procedure.

In this study, the ESEM imaging technology was used to evaluate the microstructure of silty clay and sand soil. The device of ESEM is FlexSEM 1000, which can directly observe the unsaturated soil with moisture. By installing a cold table, the temperature control range of the device can be set between − 25 and 50 °C. The experimental scheme of the soil samples is presented in Table 2. According to the test scheme, soil samples with different moisture content were prepared and sealed for 12 h. The actual moisture content of the soil samples was then measured using the drying method. First, the cutting ring sample (diameter: 61.8 mm, height: 40 mm) with a compaction degree of 100% was prepared using a self-designed sample preparation instrument. Next, the cutting ring sample was broken to extract a central soil sample (about 5 mm × 5 mm × 2 mm) for observation, ensuring the freshness and accuracy of the moisture content. The device of ESEM was set to an acceleration voltage of 5.00 kV, electron gun 26 uA, SPOT 40, and used to observe the microstructural characteristics of studied soil under different moisture content and temperature conditions. To ensure the relatively complete and averaged representation of particle and pore information in the ESEM image, there magnifications (× 100, × 1000, × 3000) were selected for observation to avoid the poor representation of quantitative data due to too small or too large scanning areas (Fig. 3).

**Table 2:** Experimental scheme of ESEM test.

Soil type	Designed moisture content (%)	Measured moisture content (%)	Measuring temperature (°C)
Silty clay	0	0.9	5, 20
	15	15.9	
	20	20.3	
Sand	0	0.5	
	9	8.8	
	15	14.5

### Matrix suction test

**Figure 4:**
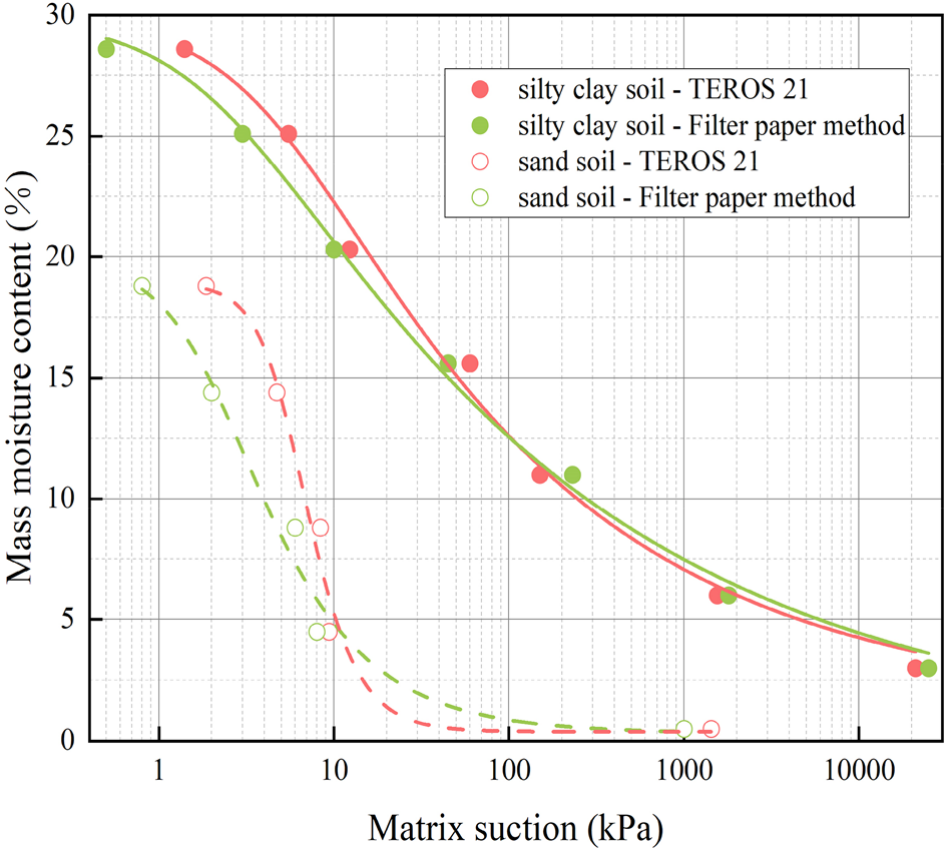
SWCC of studied soil at different test method.

The non-isothermal matrix suction test apparatus was a TEROS 21 moisture potential meter produced by the US. METER companies, which consisted of five soil moisture potential sensors and an Em50 moisture potential collector. The test range of matrix suction, and temperature measurement was – 1 kPa ~ − 100,000 kPa with the accuracy of 1 kPa, and − 40 °C–60 °C with the accuracy of 1 °C, respectively. And the test balance time was 60 min. In order to verify the accuracy of the TEROS 21 moisture potential meter, the matrix suction of silty clay and sand measured by filter paper method and TEROS moisture potential meter method at 25 °C were compared. The SWCC was fitted by Van Genuchten (VG) model^23^, as shown in Fig. 4 and Table 3. And the fitting parameters of the VG model are shown in Table 3.

**Table 3:** SWCC fitting parameters at different test method.

Soil type	Temperature (℃)	Test method	${\uptheta }_{\mathrm{s}}$ (%)	${\uptheta }_{\mathrm{r}}$ (%)	$\mathrm{\alpha }$ (kPa^−1^)	n	$\mathrm{m}$	R^2^
Silty clay	25	TEROS 21	29.86	1.40	0.225	1.2979	0.2295	0.9883
		Filter paper method	29.86	0	0.458	1.2258	0.1842	0.9939
Sand		TEROS 21	18.41	0.40	0.138	5.50	0.8181	0.9580
		Filter paper method	19.93	0.33	0.468	1.94	0.4845	0.9253

1 $$\theta (\Psi )={\theta }_{r}+\frac{{\theta }_{s}-{\theta }_{r}}{{\left[1+{\left(\alpha \Psi \right)}^{n}\right]}^{m}}$$

where $\theta (\Psi )$ is the soil moisture content (cm^3^ cm^−3^), ${\theta }_{r}$ is the residual soil moisture content (cm^3^ cm^−3^), ${\theta }_{s}$ is the saturated soil moisture content (cm^3^ cm^−3^), $\Psi$ (kPa) is the soil matrix suction, $\alpha$ (kPa^−1^) is a fitting parameter related to the inverse of air entry pressure, $n$ (–) is a parameter associated with the soil pore distribution, and $m=1-1/n$.

Comparing the test results of the filter paper method and the TEROS 21 water potential meter, it was found that the matrix suction data measured by the two methods were relatively close. It showed that the method of testing soil water characteristic curve by TEROS 21 water potential instrument proposed in this paper was reasonable.

**Figure 5:**
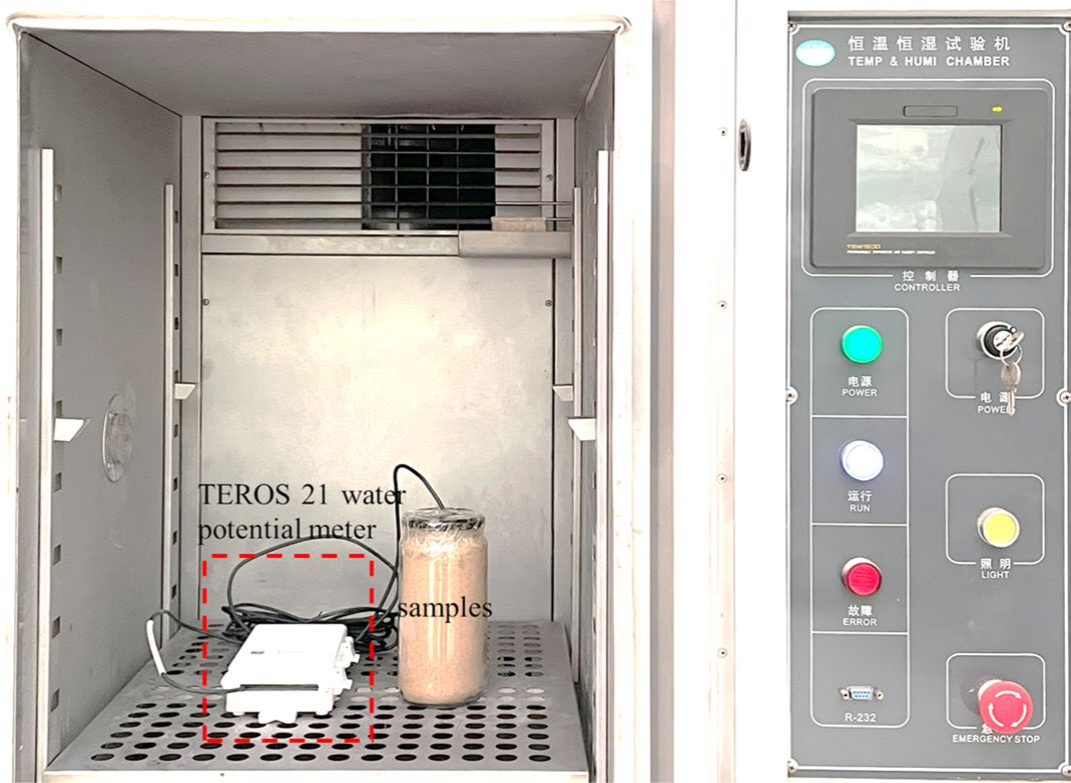
Matrix suction test process.

This experimental scheme of the soil samples is presented in Table 4. Firstly, the TEROS 21 soil moisture potential sensor was embedded in a container containing soil samples, and the other end was connected with the Em50 moisture potential collector. Next, the samples were put into the thermostat, as shown in Fig. 5. In order to ensure the accuracy of the results, three parallel specimens were tested, and the measurement deviation was less than 5%.

**Table 4:** Matrix suction test design.

Soil type	Designed moisture content (%)	Measured moisture content (%)	Measuring temperature (°C)
Silty clay	0	0.9	0, 10, 15, 20, 25, 30, 35, 40, 45
	5	6.0	
	10	11.0	
	15	15.9	
	20	20.3	
	25	25.1	
	30	28.6	
Sand	0	0.5	
	5	4.5	
	9	8.8	
	15	14.5	
	19	18.8

## Experiment results

### Temperature and moisture variation of soil column

#### Temperature field variation

**Figure 6:**
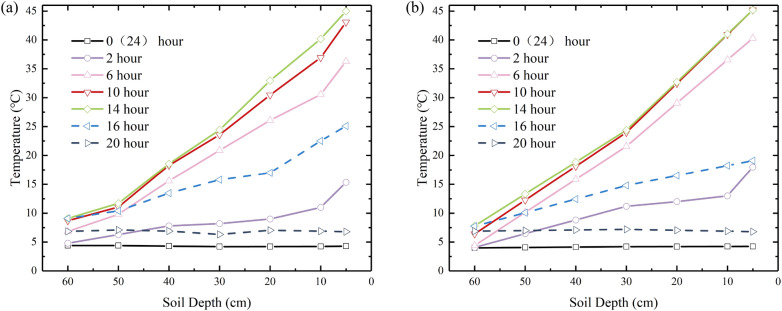
Diurnal temperature variation of soil column: (**a**) Silty clay; (**b**) Sand.

The diurnal temperature variation for the silty clay and sand soil columns is seen in Fig. 6. The diurnal temperature variation of the silty soil column was similar to sand. Because the specific heat capacity for silty clay was larger than that for sand, the rate of temperature change for silty clay was less than that for sand.

As an example, the temperature variation of a column of silty clay soil was analysed. Before turning on the light, the entire soil column had a temperature of around 4.2 °C, similar to the room of constant temperature. After turning on the light, the temperature of the upper soil increased rapidly and reached a maximum after 14 h, about 45 °C. Meanwhile, the temperature of the lower soil gradually increased accompanied by the transfer of temperature. Due to the hysteresis of heat transfer, the lower layers of the soil are significantly less affected by heat sources. After the lights are turned off, the temperature of the impermeable lid drops sharply to the same temperature as a room at a constant temperature. And the impermeable lid was like a “cold source”. Meanwhile, the soil column temperature continued to decrease under the influence of the “cold source”. After 10 h, the temperature of the soil column was reduced to the same temperature as the room at a constant temperature. Under the control of light, the temperature field variation in the soil column is similar to the temperature variation in the actual road engineering^21,24^.

#### Moisture field variation

**Figure 7:**
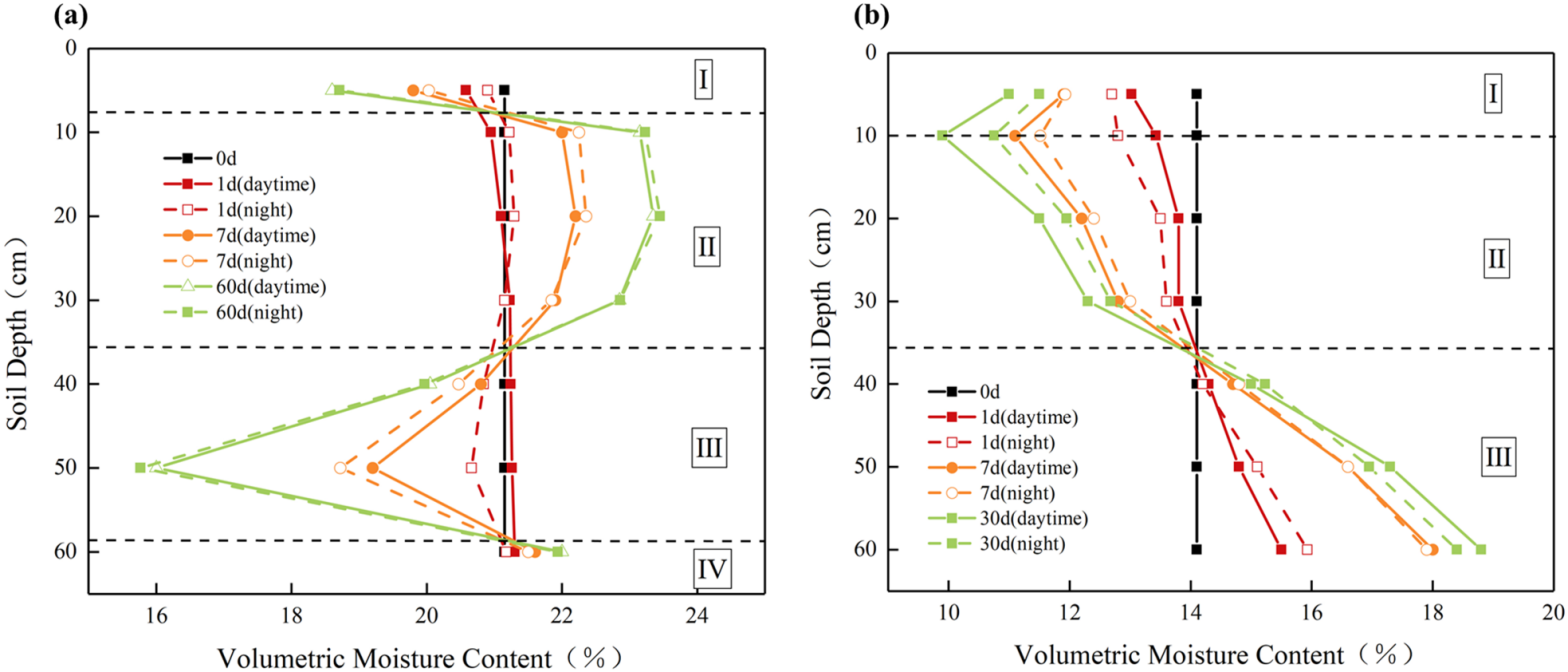
Moisture distribution of soil column: (**a**) Silty clay; (**b**) Sand.

The moisture distribution of the silty clay and sand soil column is shown in Fig. 7. It can be observed from Fig. 7 that the silty clay and sand soil columns showed significant moisture migration at multiple diurnal cycles.

However, the behaviour of moisture migration in silty clay and sand soil showed a clear difference. In the case of the silty clay soil column (Fig. 7a), the moisture content gradually decreased in region I (0–8 cm) and region III (38–58 cm) over time compared to the initial moisture content, while the moisture content in region II (8–38 cm) and region IV (58–65 cm) gradually increased. After 60 days of testing, the maximum moisture content was found to be 23.45% at a depth of 20 cm. Moisture migration in the silty clay soil column occurs primarily upwards. As for the sand soil column (Fig. 7b), the moisture content in region I (0–10 cm) and region II (10–38 cm) decreased gradually during the test, whereas the moisture content in region III (38–65 cm) increased. The moisture in the sand soil column mainly moved downward. At a depth of 10 cm, there is an inflection point in the moisture distribution, where the moisture content in the soil layer at a depth of 0–10 cm is higher than at 10 cm. Zhang et al. simulated the moisture migration process in subgrade soils under the influence of pavement structure’s blocking effect and seasonal temperature, in which moisture distribution was similar to the moisture distribution of silty clay and sandy soil columns in this paper^9^.

**Figure 8:**
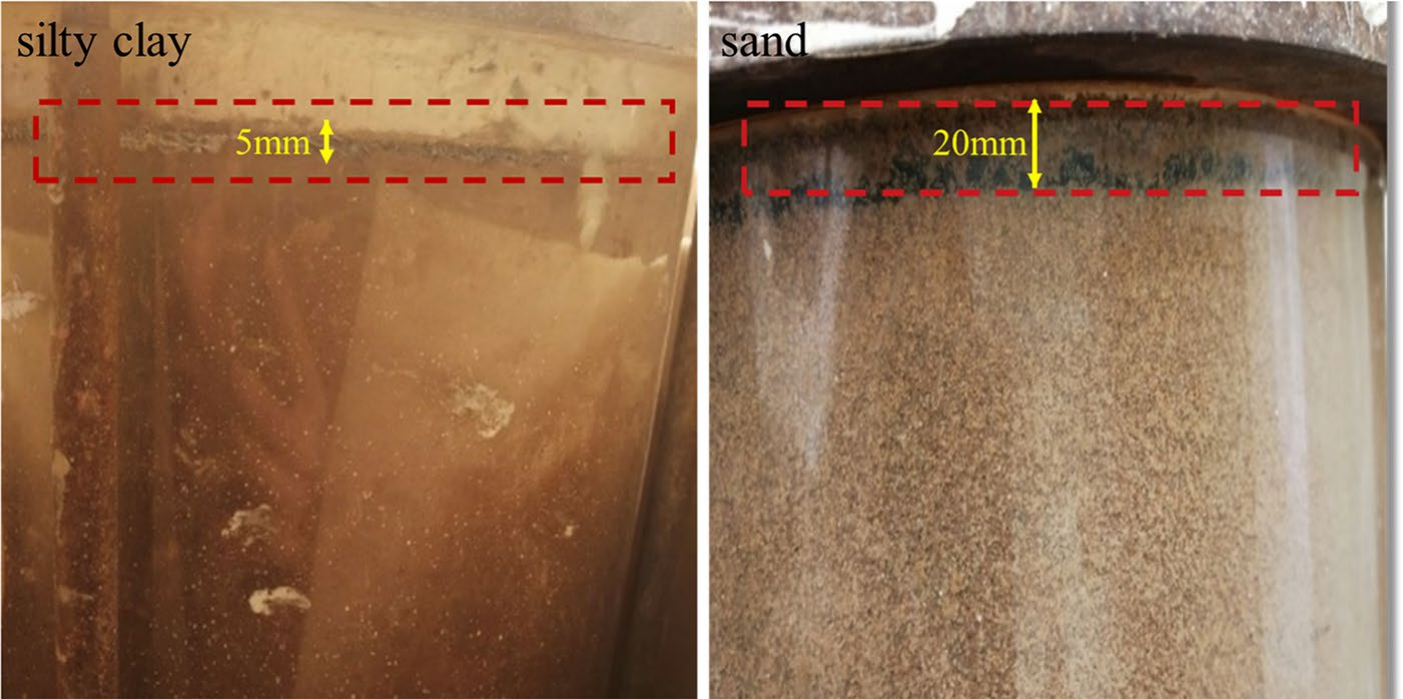
Soil column shrinkage.

By comparing the daytime and nighttime moisture fields in the same cycle, it was observed that moisture in the lower soil layer of the silty clay soil column migrated to the upper soil layer at night. For the sand soil column, at the start of the experiment (1 day), moisture in the upper soil layer was observed to migrate to the lower soil layer at night. During other testing periods, however, moisture from the lower soil layer migrated to the upper soil layer. In addition, during testing, it was found that the upper soil layer of the silty clay and sand soil column shrank, forming a cavity between the covering layer and the soil column, as shown in Fig. 8. During the night, large droplets of liquid water form at the base of the covering. A large number of liquid water droplets formed in the bottom of the covering layer during the night. During the night, large droplets of liquid water form at the base of the covering. Since liquid water can hardly migrate through the gap to the bottom of the lid, this proves that the droplet originates from vapor moisture migration. Similar phenomena such as field experiments were carried out in arid areas, and it was concluded that plastic film mulching prevented water vapor evaporation loss and increased soil moisture storage in the upper layer^25,26^.

### Microstructure characteristics

**Figure 9:**
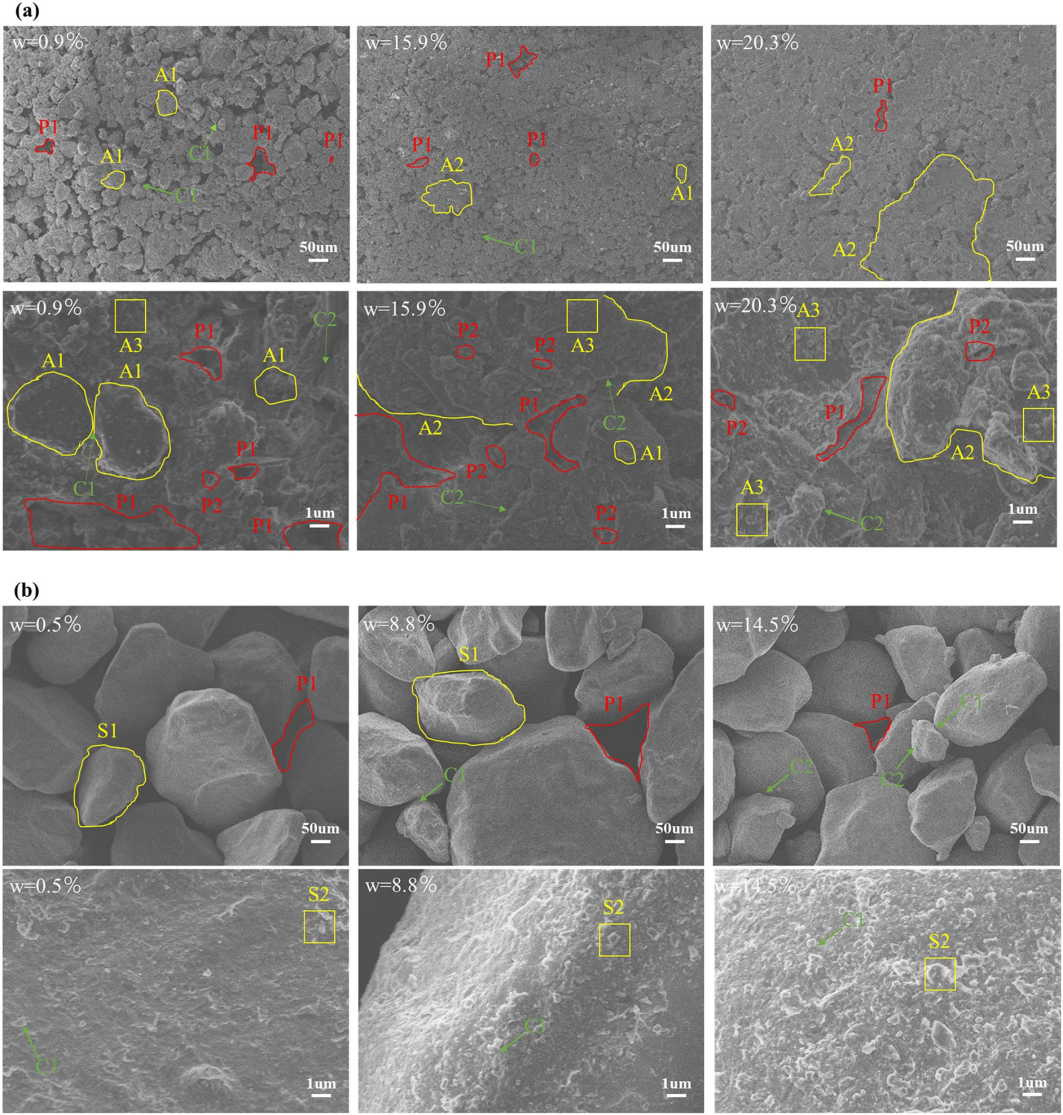
Microstructure morphology of soil samples at two magnifications: (**a**) silty clay; (**b**) sand. A1 silty grain, A2 silty aggregate, A3 clay grain, S1 sand grain, S2 small sand grain; C1 point connection, C2 surface connection; P1 inter-aggregate pore, P2 intra-aggregate pore.

Figure 9 shows the ESEM images of silty clay and sand soil at two different magnifications: × 100 and × 3000. The microstructure of the soil sample at × 100 magnification provided a wider field of view, while the hydration state and micropore structure of the soil sample at × 3000 magnification were clearer. Therefore, for the purpose of preserving the integrity of microstructure information, the microstructure images captured at × 100 magnification were selected for further quantitative analysis. By combining the images at × 3000 magnification, changes in the hydration state and micropore structure were observed.

**Figure 10:**
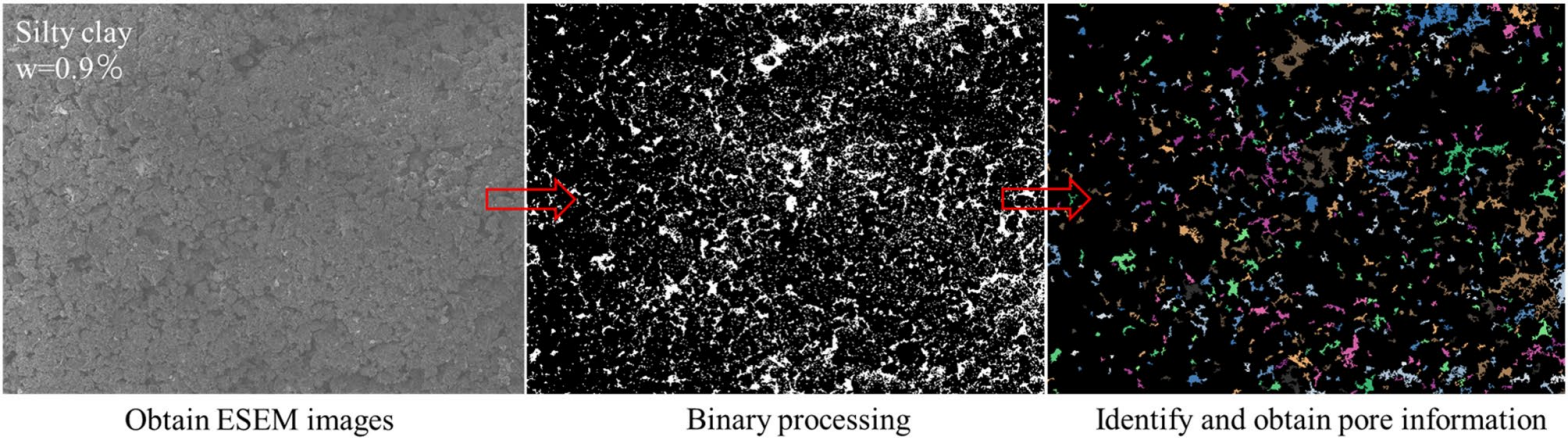
Binarize of ESEM images.

In general, the original image obtained cannot be directly used for quantitative analysis. It is necessary to enhance, segment, remove noise, and then binarize the image. This paper utilized the PCAS software to binarize the ESEM images, in order to derive the geometric and statistical parameters of the pores, including parameters such as the number, area, perimeter, length, and width. Taking the binarization processing of silty clay image with a concentration of 0.9% as an example, the process is presented in Fig. 10. In addition, this study introduced Lei’s pore classification method to classify pores into four types according to pore diameter, namely macropores larger than 32 um in diameter, mesopores between 8 and 32 um in diameter, small pores between 2 and 8 um in diameter, and micropores less than 2 um in diameter^27^.

**Figure 11:**
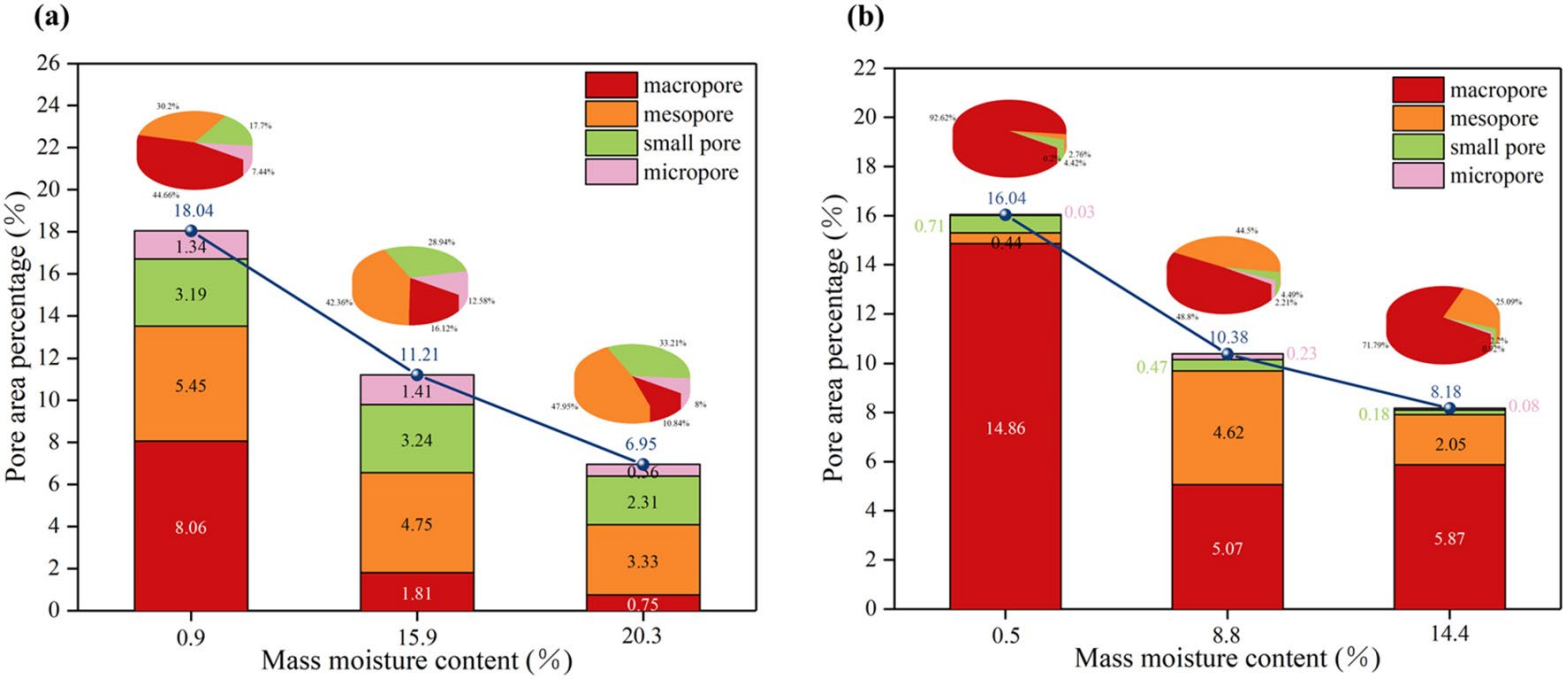
Area distribution changes of four kinds of pores: (**a**) silty clay; (**b**) sand.

From Fig. 9a at a × 100 magnification, it is evident that the silty grain (A1) of silty clay (w = 0.9%) is small and independent, with a porosity of 18.04%. The inter-aggregate pores (P1) are numerous and evenly distributed, with a predominance of macropores and mesopores, as shown in Fig. 11. As the moisture content increases to 15.9%, the particles start to agglomerate into silty aggregate (A2), resulting in a decrease in the porosity to 11.21% and a reduction in the inter-aggregate pores (P1). The percentage of macropores decreases significantly from 8.06% to 1.81%, while small pores and micropores show a slight increase. Yuan et al. also used ESEM and observed that with increasing moisture content, the aggregate size of compacted silty clay gradually increased^28^. With further increase in moisture content to 20.3%, the particles continue to agglomerate, forming even larger particles, causing a continuous decrease in the inter-aggregate pores (P1) and a decrease in porosity to 6.95%. The proportion of mesopores decreases significantly, from 4.75 to 3.33%. In the image magnified to × 3000, the silty clay (w = 0.9%) is primarily comprised of large silty grains (A1), while small silty grains (A1) are connected to the large silty grains through point (C1). Additionally, scattered small clay grains (A3) adhere sporadically to the surface of the silty grain (A1). The inter-aggregate pores (P1) were more. When the moisture content increases to 15.9%, these small silty grain particles (A1) gather together to form silty aggregates (A2). Concurrently, the small clay grains (A3) become moisture-filled and attach to the surface of the aggregates (A2), forming clay-silty aggregates. Since the soil sample (w = 15.9%) is closer to its optimum moisture content (w = 14.1%), it becomes easier to compact, with the surface connection (C2) between particles becoming the main form of connection. The inter-aggregate pores (P1) decrease, while the intra-aggregate pores (P2) increase. As the moisture content continues to rise to 20.3%, more small clay grains (A3) adhere to the surface of the aggregates (A2), resulting in the clay-silty aggregates becoming the primary constituents of the particles, and the inter-aggregate pores (P1) and intra-aggregate pores (P2) further reducing.

It can be observed in Fig. 9b that under × 100 magnification, as the moisture content increases, sand grains (S1) move closer to each other due to surface tension, leading to a gradual decrease in porosity. Unlike silty clay, sand lacks cohesion and therefore does not form large grains. The porosity decreases from 16.04 to 10.38% as the moisture content increases from 0.5 to 8.8%. The proportion of macropores is significantly reduced and transformed into mesopores. When the moisture content reaches 14.4%, the porosity further decreases to 8.18%. The fraction of macropores slightly increases and the fraction of mesopore areas decreases significantly. In the × 3000 magnification image, as the moisture content increases, small sand particles (S2) become filled with moisture and are increasingly adsorbed onto the surface of large sand grains through point-to-point contact (C1). Additionally, the pores primarily consist of inter-aggregate pores (P1).

Quantitative analysis reveals that silty clay primarily contains mesopores, while sand contains mainly macropores. The average pore size of sand is larger than that of silty clay.

**Figure 12:**
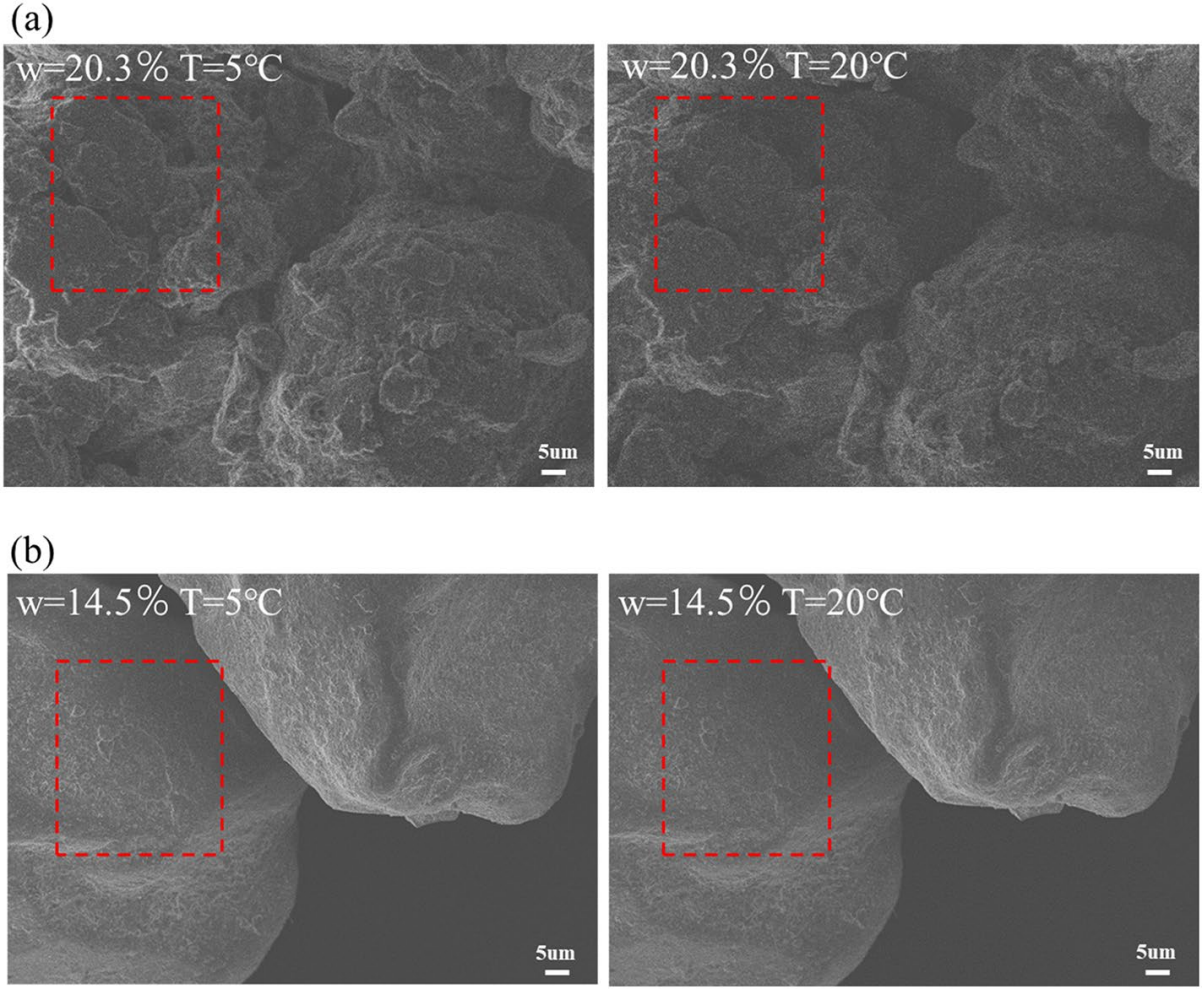
Microstructure characteristic of soil sample at different temperature: (**a**) silty clay; (**b**) sand.

Additionally, it is found from Fig. 12 at a × 1000 magnification that the surface texture of silty clay and sand at 5 °C is clearer than that at 20 °C, indicating that as the temperature increases, the moisture film becomes thicker and the surface texture becomes blurred. G. Montes-H and Lin et al. used ESEM to observe the hydration/dehydration cycle of bentonite MX80 and shale weathered expansive soils and also found that the moisture film of studied soil became thicker under the action of hydration^16,17^.

In order to quantify the orientation distribution and homogeneity of pores in silty clay and sand, probability entropy (*H*) and fractal dimension (*Df*) are introduced in this study^18^. The calculation principle is as follows:

The actual pore area (*S*) and the actual perimeter (*C*) are converted by Formula (2) and Formula (3).

2 $$S=s/{R}^{2}$$

3 $$C=c/R$$

where *S* and *C* are the actual pore area and the actual pore perimeter, respectively, *s* and *c* are the pore pixel area and the pore pixel perimeter, respectively, and *R* is the resolution of the electron microscope image (pixels/µm).

Probability entropy (*H*) represents the direction of pore expansion, and the value range of *H* is 0 ~ 1. The larger *H* means the more disordered the arrangement of pores and the worse the orientation, which can be obtained by Formula (4).

4 $$H=-\sum_{i=1}^{n}{P}_{i}{\cdot log}_{n}{P}_{i}$$

where *P*_*i*_ is the probability of the pore in this direction angle.

The fractal dimension (*Df*) reflects the pore morphological complexity, and the value range of *Df* is 1 ~ 2. A large fractal dimension indicates a more complex pore morphology and a more undulating particle interface, which can be obtained by Formula (5).

5 $$log\left(C\right)=Df/2\cdot log\left(S\right)+{c}_{1}$$

where *Df* is the fractal dimension and c_1_ is a constant.

It can be seen from Table 5 that the probability entropy (*H*) and fractal dimension (*Df*) of silty clay and sand fluctuate with changes in moisture content. Nie et al. obtained similar results through studying mechanical behaviour and microstructural variation of loess under dynamic compaction^29^, and concluded moisture content has no significant effect on the complexity of pore morphology. The reason for this is that pore morphology depends heavily on the morphology and arrangement of particles. However, changes in moisture content alone have little effect on soil particle morphology and have difficulty significantly affecting the development or inhibition of more complex pores. The probability entropy of silty clay ranges between 0.950 and 0.963, while the probability entropy of sand ranges between 0.687 and 0.867. A higher probability entropy indicates a stronger randomness in pore development direction, making it more difficult to form connected pores. The fractal dimension of silty clay ranges between 0.986 and 1.152, while the fractal dimension of sandy soil ranges between 0.738 and 1.066. A higher fractal dimension indicates a stronger heterogeneity of pores and greater tortuousness of pore channels^18^. Based on the above analysis, compared with silty clay, the pore channels of sand are more connected and less tortuous, which is conducive to moisture migration.

**Table 5:** Probability entropy and fractal dimension of pore.

Soil type	Silty clay			Sand		
Moisture content (%)	0.9	15.9	20.3	0.5	8.8	14.5
Probability entropy	0.9588	0.9629	0.9508	0.8670	0.8302	0.6873
Fractal dimension	1.1075	0.9868	1.1512	0.8456	0.7382	1.0653

### Thermal effect of soil–water characteristic curve

**Figure 13:**
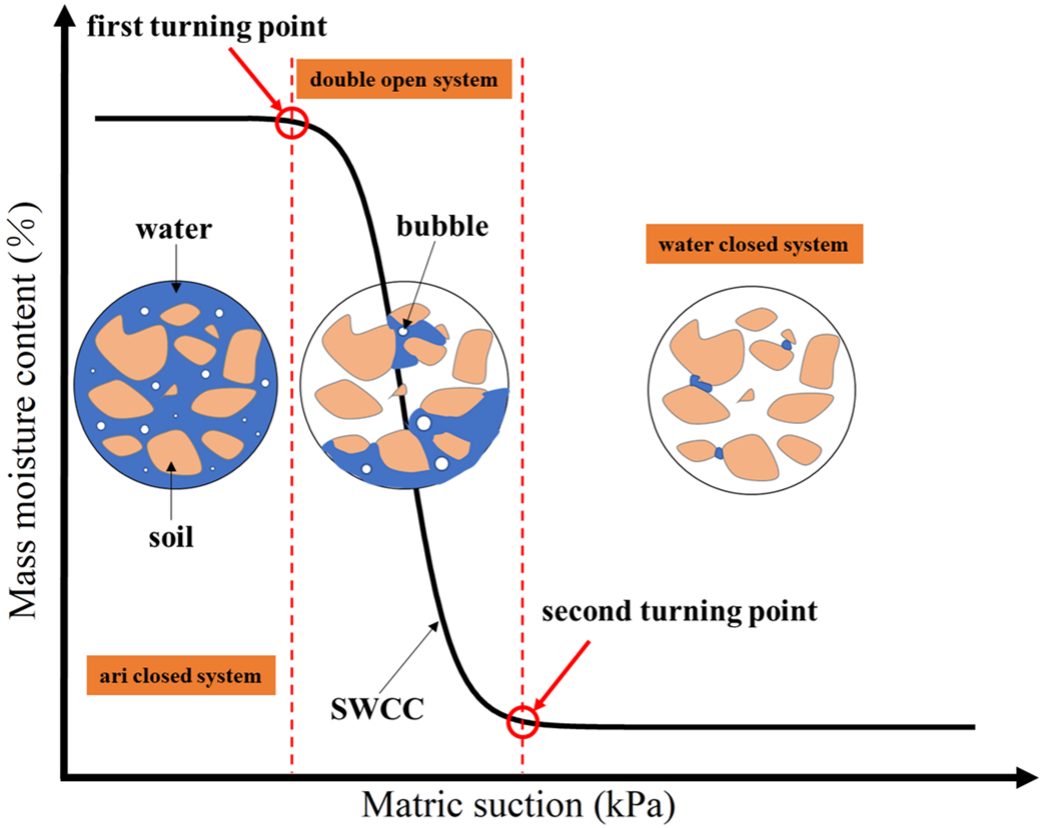
Pore structure system of unsaturated soil.

The change characteristics of SWCC were analysed from the perspective of soil microstructure. As shown in Fig. 13, unsaturated soil structure can be classified into three categories based on the state of moisture and gas in soil pores: water-closed, double-open, and gas-closed. When the matrix suction is less than the air entry value of the soil, the pores are filled with liquid water and the gas remains in the form of isolated bubbles, which is called the gas-closed state. In this state, the moisture migration happens through liquid water migration. As the matrix suction exceeds the air entry value, the liquid water in the large pores starts to discharge and the first turning point appears on the SWCC. When the matrix suction continues to increase, two channels of liquid and gas are formed in the pores, both of which are connected to the surface of the soil, which is called the double-open system. Moisture migration in the soil is a combination of liquid–vapor migration in this state. When the matrix suction is large enough, most of the free water in pores will be discharged and the SWCC reaches the second turning point, which enters the residual water content state. At this point, the liquid water is not enough to connect with each other, and most of the pores are occupied by the gas phase, forming a liquid water closure state. Moisture migration mainly happens through water vapor migration.

**Figure 14:**
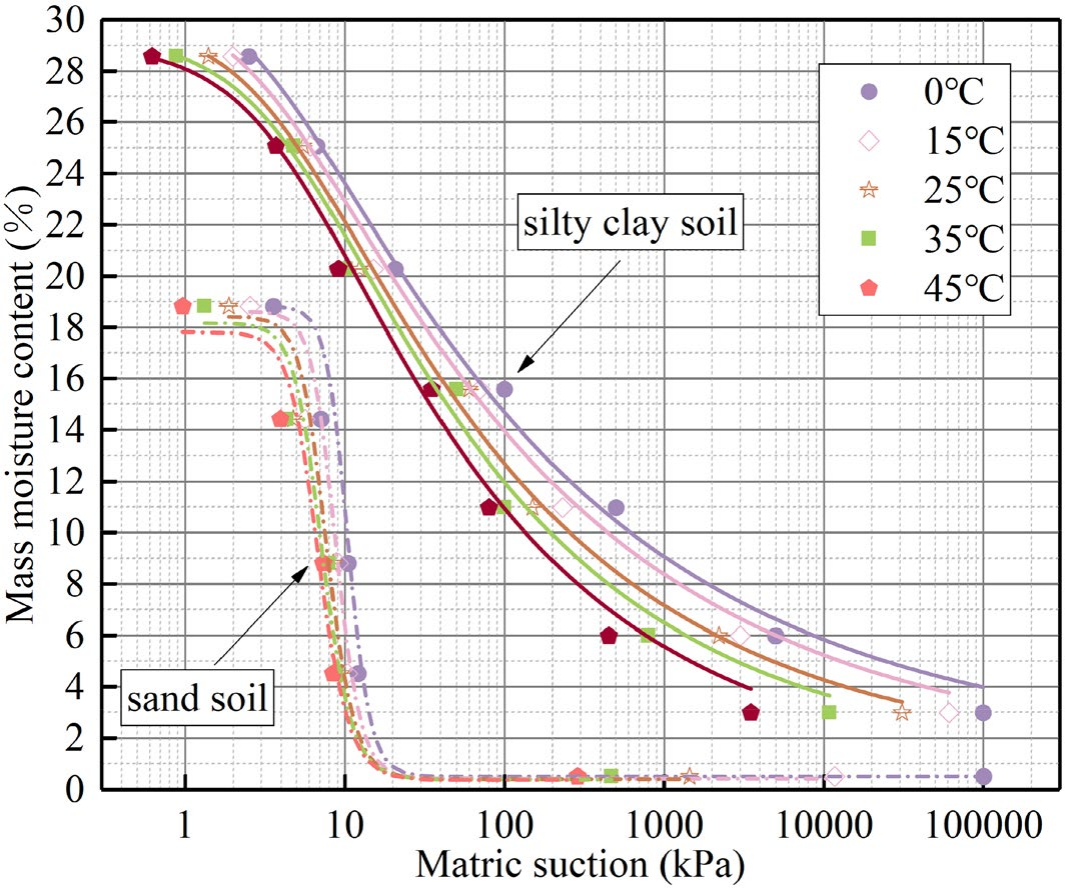
SWCC at different temperature.

The SWCC of the studied soil at non-isothermal condition was fitted by the VG model based on the results of matrix suction test, as shown in Fig. 14. The fitting parameters of the VG model are shown in Table 6.

**Table 6:** SWCC fitting parameters at different temperature.

Soil type	Temperature (°C)	${\theta }_{s}$ (%)	${\theta }_{r}$ (%)	$\alpha$ (kPa^−1^)	*n*	$m$	*R*^*2*^
Silty clay soil	0	30.10	2.00	0.150	1.2748	0.2155	0.9832
	15	29.91	1.50	0.200	1.2862	0.2225	0.9932
	25	29.86	1.40	0.225	1.2979	0.2295	0.9883
	35	29.55	0.90	0.257	1.3000	0.2307	0.9813
	45	29.36	0.50	0.260	1.3290	0.2395	0.9859
Sand soil	0	18.81	0.50	0.105	6.60	0.8485	0.9410
	15	18.62	0.42	0.121	6.00	0.8333	0.9611
	25	18.41	0.40	0.138	5.50	0.8181	0.9580
	35	18.26	0.38	0.148	5.20	0.8076	0.9523
	45	17.79	0.36	0.157	5.00	0.8000	0.9673

It can be seen from Fig. 14 that the SWCC of silty clay is located above that of sand. The reason for this phenomenon can be analysed as follows: according to the Young–Laplace formula^30^ (Formula 6), the matrix suction of unsaturated soil is inversely proportional to the pore radius of the soil. The average pore radius of silty clay is smaller than that of sand. Consequently, at the same moisture content, the matrix adsorption of silty clay is significantly larger than that of sand.

6 $$\Psi =\frac{2{T}_{S}cos\alpha }{r}$$

where ${T}_{S}$ is surface tension of moisture, $\alpha$ is contact angle, *r* is average pore radius of soil.

At the same time, the SWCC moves to the lower left as the temperature increase, and the two turning points of the SWCC gradually decrease. Zhang^13^, Wilkinson^31^, Grant^30^ et al. confirmed the thermal effect of matrix suction through laboratory experiments. The reason is that, for the gas-closed state, the gas has some solubility in liquid water and has a strong thermal expansion. As the temperature increases, the gas dissolved in the liquid water expands and the liquid water in the pores is squeezed out. As a result, soil with the same matrix suction and low temperature has a larger water-holding capacity than soil with high temperatures. The moisture in the water-closed state is dominated by bound water. The thickness of the bound water is temperature dependent and the amount of adsorbed bound water decreases with increasing temperature. When the temperature is increased, the motion of bound water molecules is intensified. When the adsorption force of the electronegative molecules is not sufficient to bind the water molecules, the outer bound water breaks free from the bonds and becomes free water, which is expelled by the suction of the substrate. The double open pore system has both bound and free water. The ESEM images also confirm that the water-holding capacity of the soil decreases and the water film of the soil becomes thicker as the temperature increases.

**Figure 15:**
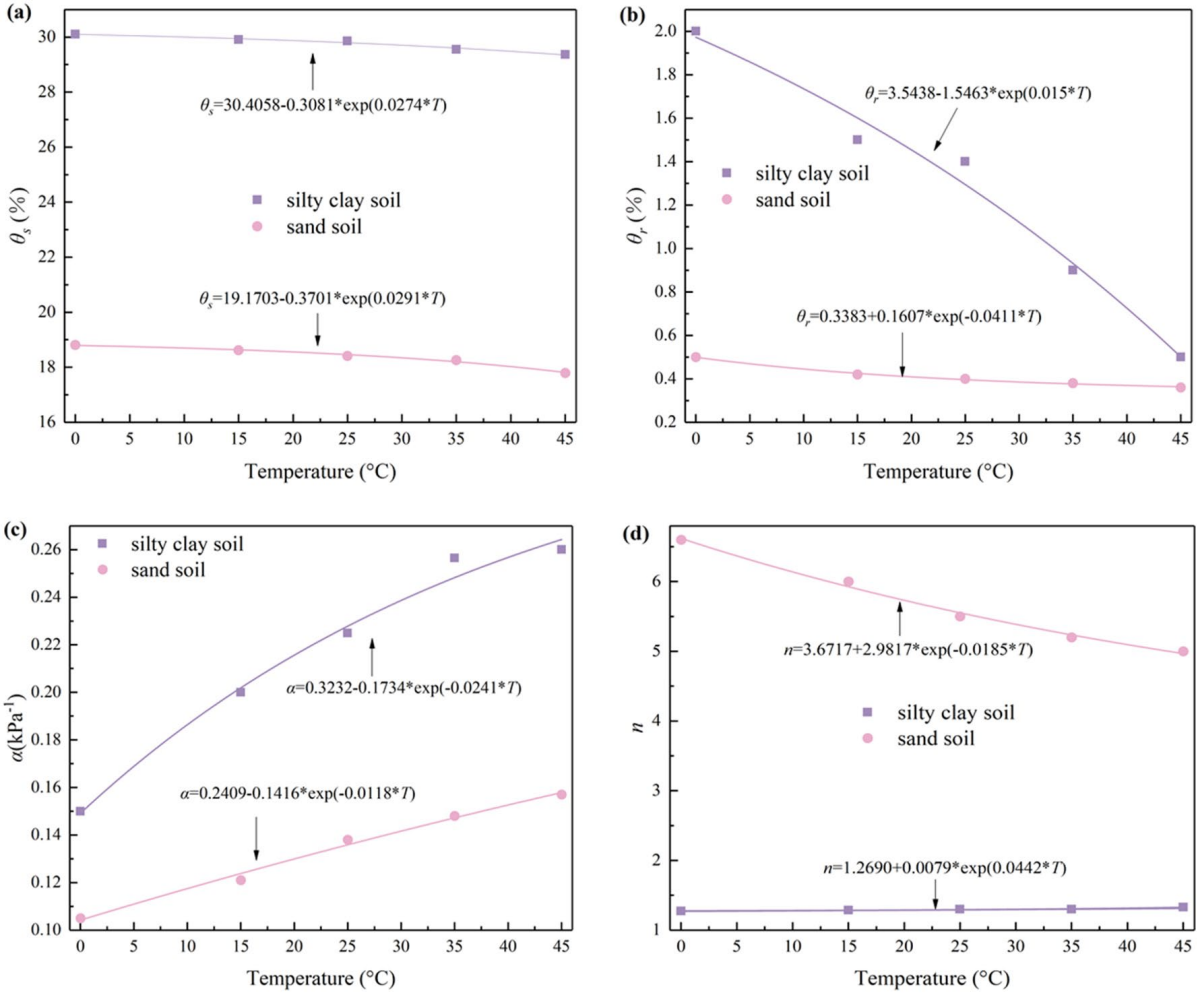
Fitting parameters and temperature.

In addition, in order to quantitatively describe the effect of matrix suction on moisture migration, the SWCC model at non-isothermal conditions describing the relationship between matrix suction, moisture content, and temperature needs to be modified. As shown in Table 6, the fitted parameters vary monotonically with temperature. An exponential function of the fitting parameters versus temperature was fitted according to Table 6, as shown in Fig. 15. Subsequently, the exponential relationship equation of each parameter with temperature was substituted into the VG model to obtain the non-isothermal SWCC model for silty clay and sand soil, as Formulas (7) and (8), respectively.

7 $$\theta =3.5438-1.5463{e}^{0.015T}+\frac{\left(26.732-0.1667{e}^{0.0444T}+1.5463{e}^{0.015T}\right)}{{\left[\frac{1}{{1+\left(\left(0.3216-0.21{74e}^{-0.0063T}\right)\Psi \right)}^{1.2093+0.0603{e}^{0.0115T}}}\right]}^{\left(1-1/\left(1.2093+0.0603{e}^{0.0115T}\right)\right)}}$$

8 $$\theta =0.3383+0.16{07e}^{-0.0411T}+\frac{\left(18.832-0.3701{e}^{0.0291T}-0.1607{e}^{-0.0411T}\right)}{{\left[\frac{1}{{1+\left(\left(0.2409-0.1416{e}^{-0.0118T}\right)\Psi \right)}^{3.6717+2.9817{e}^{-0.0185T}}}\right]}^{\left(1-1/\left(3.6717+2.9817{e}^{-0.0185T}\right)\right)}}$$

**Figure 16:**
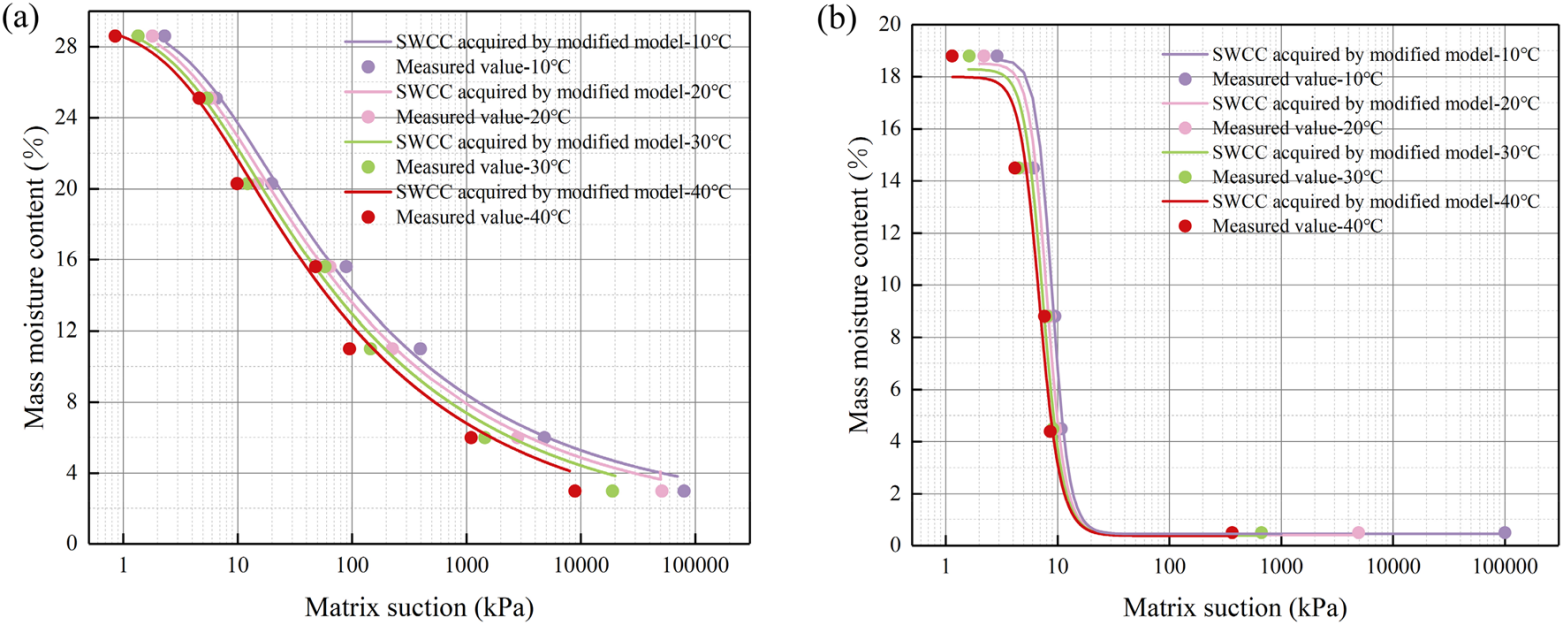
Comparison of SWCC obtained by Modified model and measured matrix suction data. (**a**) Silty clay soil; (**b**) Sand soil.

To validate the reasonableness of the modified SWCC model, the soil–water characteristic curves at 10, 20, 30, and 40 °C were calculated using the non-isothermal SWCC model and compared with the measured values of matrix suction test, as shown in Fig. 16. The SWCC and measured matrix suction data are highly fitting, which proves that the modified SWCC model at the non-isothermal condition for sand soil and silty clay soil is rational.

## Discussion

**Figure 17:**
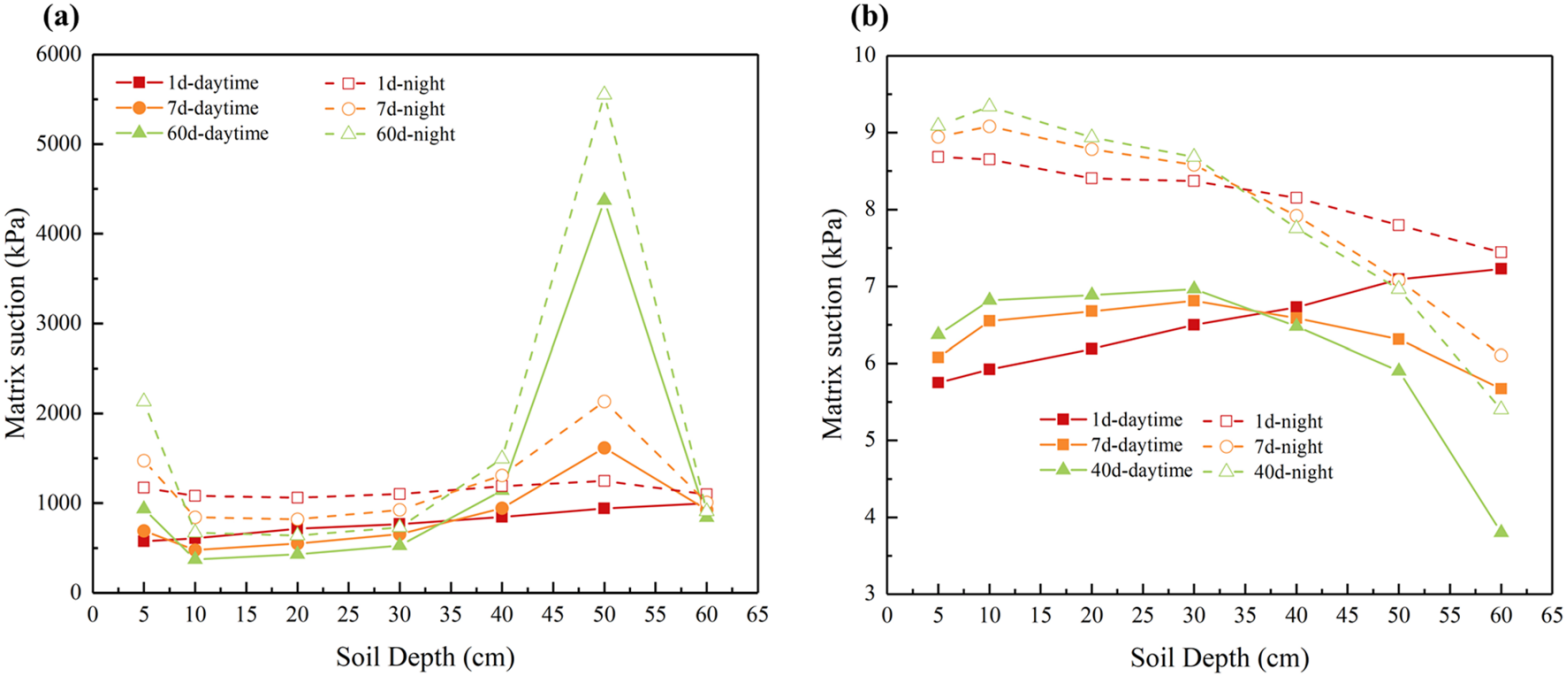
Matrix suction of soil column varying with time.

Moisture migration in unsaturated soils was a result of the interaction between temperature and soil microstructure. The above results indicate that this was a liquid–vapor mixing migration for silty clay and sand soil columns. The diurnal temperature difference affected the moisture migration process by altering the moisture potential energy of the soil microstructure. Liquid water was mainly migrated by matrix potential and gravity potential, while vapor moisture was migrated by moisture vapor pressure gradient^23^. Based on the temperature field and moisture field, Formulas (7) and (8) were employed to calculate the changes in matrix suction of silty clay and sand soil with time, as shown in Fig. 17. At the same time, the water vapor pressure of the soil was calculated using the Kelvin formula, and it was found that the water vapor pressure is mainly affected by the change in temperature and less by the suction of the matrix. Therefore, the change in water vapor pressure only considers the effect of diurnal temperature variations. The gravitational potential is proportional to the height of moisture in the soil. In addition, the moisture migration process is analysed based on the effect of temperature on the moisture potential energy.

Figure 17a demonstrates that, for silty clay, the matrix suction in the upper soil layer is generally smaller than that in the lower soil layer. As a result, liquid water migrated downward under the influence of both the matrix and gravitational potentials during the daytime and night. Regarding vapor moisture, during the daytime, heat was transferred from the top to the bottom, leading to a greater pressure of water vapor in the upper soil layer compared to the lower soil layer. As a result, the water vapor migrated downward due to the water vapor pressure. At night, the covering layer at the top of the soil column rapidly cooled down and became a “cold source”. Water vapour from the lower soil layer migrates upward to the covering layer and condenses into liquid water, replenishing the moisture in the upper soil layer due to the gravitational potential. In this paper, this phenomenon, named the “diurnal cycle of water vapor migration”, includes the migration downward of water vapor during the daytime, the upward migration and condensation at night under the influence of the “cold source”, and the subsequent downward recharge under the action of gravity potential. In the study of water vapor flow, many scholars also pointed out that water vapor will change regularly with daily cycle and seasonal cycle^23,32^. Notably, region I (0–8 cm) in the silty clay soil column was closer to the heat source, resulting in greater moisture migration during the daytime compared to the water vapor supply from the lower soil at night. Thus, the moisture content in region I (0–8 cm) gradually decreased with time. However, the moisture content in region II and region III was primarily supplied by the water vapor from the lower soil at night, leading to a gradual increase in moisture content. In addition, for silty clay, it has been confirmed that a high matrix suction will greatly reduce the permeability coefficient of liquid water, and inhibit the migration of liquid water^33^.

From Fig. 17b, it can be observed that the sand soil column also appeared in the phenomenon of the “diurnal cycle of water vapor migration”. As a result, the moisture distribution in region I (0–10 cm) of the sand soil column showed a non-monotonic feature. However, in contrast to silty clay, sand has a smaller suction of the matrix, leading to a dominant downward migration of moisture under the influence of the gravitational potential.

**Figure 18:**
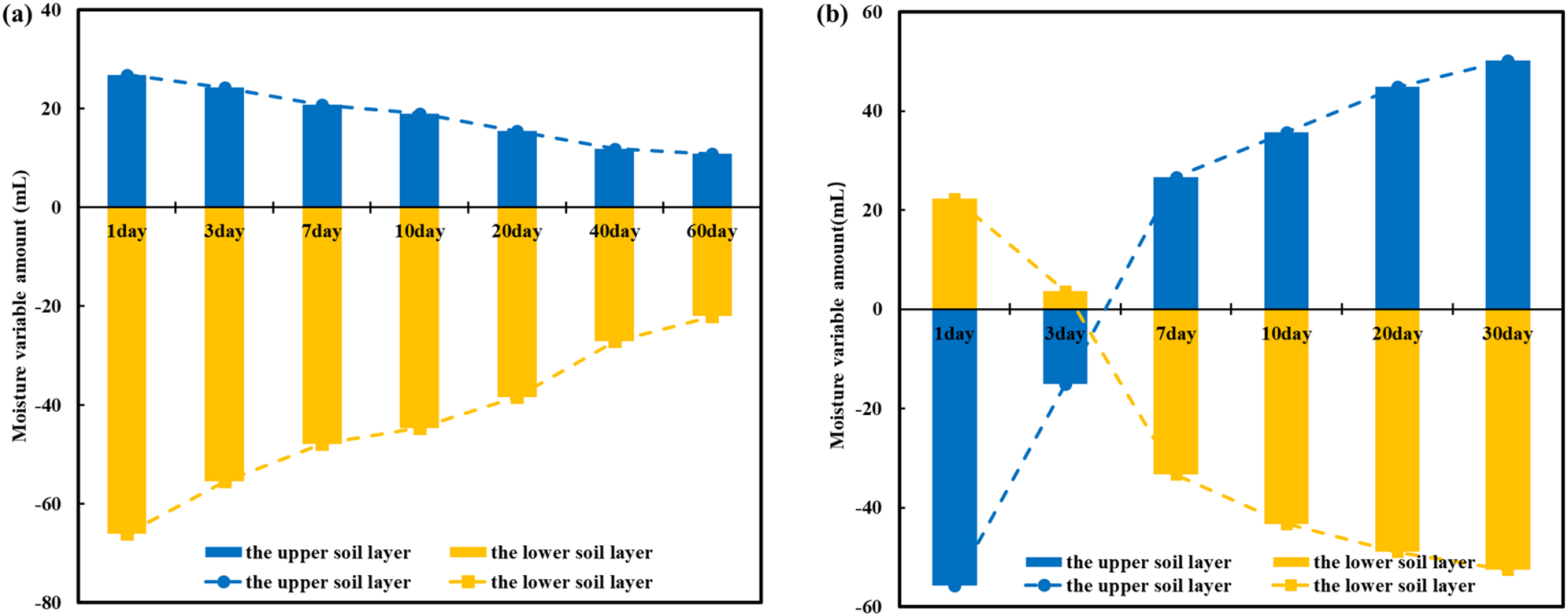
Amount of moisture migration at night varies with times: (**a**) silty clay; (**b**) sand.

The mechanism of the moisture distribution was revealed by analyzing the changes in the water potential energy of soil microstructures. At the same time, moisture redistribution and soil microstructure affect each other in the process of moisture migration. As time progressed, the moisture redistribution affected the soil microstructure, which in turn influenced the process of moisture migration^34,35^. By integrating the moisture distribution curves of different cycle times, the moisture changes of the upper soil layer and the lower soil layer at night compared with the daytime are obtained, as shown in Fig. 18. As time goes on, the moisture migration in the upper and lower soil layers of the silty clay soil column gradually decreased. On the one hand, this was because the matrix suction in the lower soil layer gradually increased and the water-holding capacity continuously increased. On the other hand, the moisture content in the 8–36 cm soil layer of the silty clay soil column increased, which led to pores (inter-aggregate pore and intra-aggregate pore) and the moisture migration channels decreased, impeding the upward migration of water vapor. For sand soils, there was a gradual increase in moisture increment for the upper soil layer and a gradual decrease in moisture reduction for the lower soil layer. Similarly, this was due to the decrease in moisture content in the upper soil layer and the gradual increase in matrix suction, which weakened the effect of gravity potential. Moreover, the decrease in moisture content in the upper soil layer increased the pore space (inter-aggregate pore), which promoted the upward movement of water vapor.

After the above analysis, it was found that due to the extreme diurnal temperature difference cycle, the phenomenon of water accumulation appeared at the bottom of the covering layer for the silty clay and sand soil column. In addition, the moisture content of silty clay at the depth of 8–38 cm increased, while the upper soil of the sand soil column experienced a decrease in moisture content. The increase in soil moisture content made the road produce a variety of diseases. This indicated that silty clay was more sensitive to the migration of moisture vapor than sand soil under the action of multiple diurnal cycles, and it would have a greater impact on the project. Eigenbrod et al. also confirmed that with the increase of clay particles, the phenomenon of water aggregation at the bottom of the covering layer is more obvious^36^.

## Conclusions

1. The porosity of silty clay and sand decreases as the moisture content increases. The pores in Silty clay are dominated by mesopores. With the increase of moisture content, the proportion of macropores first decreases, followed by the decrease of mesopores. The pores in Sand is dominated by macropore. With the increase of moisture content, the proportion of macropore first decreases, followed by the decrease of mesopore. The probability entropy and fractal dimension of soil pore for silty clay and sand fluctuate with the change of moisture content. Compared with silty clay, sand has a pore structure that is more conducive to the formation of connected pore channels.
2. The matrix suction of silty clay and sand is inversely proportional to temperature. The ESEM image reveals that under the same moisture content condition, the higher the soil temperature, the smaller the moisture holding capacity and the thicker the moisture film. Silty clay has a greater water-holding capacity compared to sand soil. The non-isothermal soil-moisture characteristic model based on the VG model was established to describe the relationship between moisture content, temperature, and matrix suction.
3. Under diurnal cycle, both silty clay and sand soil columns appeared a phenomenon known as the “diurnal cycle of water vapor migration”, which led to moisture accumulate at the bottom of the covering layer. The overall pore structure of silty clay is small, resulting in strong water-holding capacity, which hindered liquid water migration. Water vapor pressure served as the main driving force, causing moisture to migrate upward. On the other hand, sand soil possesses the larger pore structure and weaker water-holding capacity. Gravity potential acted as the main driving force, causing moisture to migrate downward.
4. Moisture migration and soil microstructure changed interact with each other. The matrix suction of the lower soil layer in the silty clay soil column increased, and the porosity of the upper soil layer decreased, resulting in a gradual decrease in the moisture migration of the silty clay soil column at night. On the other hand, the matrix suction and porosity of the upper soil of the sand soil column increased, leading to a gradual increase in the moisture migration of the sand soil column at night.

## Data Availability

Some or all data, models, or codes that support the findings of this study are available from the corresponding author upon reasonable request (list items).
